# Effectiveness and safety of Xinyin tablet in treatment of chronic heart failure: A protocol of systematic review and meta-analysis of randomized controlled trials

**DOI:** 10.1097/MD.0000000000023759

**Published:** 2020-12-18

**Authors:** Qingqing Liu, Xi Huang, Mingyang Tian, Xiaoming Dong, Jinhua Kang, Huili Liao, Jianhong Liu, Yan Ling, Wenjie Long, Zhongqi Yang

**Affiliations:** a The First Clinical Medical College; b Department of Geriatrics, The First Affiliated Hospital, Guangzhou University of Chinese Medicine, Guangzhou, Guangdong, China.

**Keywords:** chronic heart failure, effectiveness and safety, meta-analysis, Xinyin Tablet

## Abstract

**Background:**

Xinyin Tablet (XYT) has been widely used in the treatment of CHF, Which helping to improve the clinical symptoms, enhance exercise, and even may improve the long-term prognosis of patients. However, the exact effectiveness and safety of XYT for CHF has not be comprehensively researched, so we want to generalize the effectiveness and safety of XYT for CHF through the meta-analysis, which may benefit the design of future clinical trials and provide valuable references.

**Methods:**

This protocol complies with the Preferred Reporting Items for Systematic Review and Meta-Analysis Protocols. From the inception until September 2020, a systematic and comprehensive electronic search about Relevant randomized controlled trials will be conducted in 4 English literature databases and 4 Chinese literature databases. The registration number: INPLASY2020100015. 2 investigators will be arranged to deal with the study selection and data extraction independently. The New York Heart Function Classification, traditional Chinese medicine (TCM) symptom scores, the scores of quality of life, 6-min walk distance (6MWD), etc. will be systematically measured as outcomes. At last, the data will be handled by Review Manager 5.3 and Stata 15.0.

**Results and conclusion:**

This study is hoping to provide a high-level evidence to prove the therapeutic effect of XYT on CHF, which may enhance the application of Chinese medicine.

## 1 Introduction

Chronic heart failure (CHF), the terminal stage of various heart diseases, has become a major health problem due to its increasing incidence, with approximately 38 million CHF patients worldwide.^[1–3]^ The American Heart Association (AHA)^[4]^ has found that the incidence of CHF in the total population is 1.5% to 2.0%, and the incidence in patients above 65 years of age is about 8%. Epidemiological data shows that the incidence of heart failure among adults in developed countries is 1% to 2%, which may exceed 10% in the patients over 70 years of age, and is the primary reason of sudden death in the elderly.^[5–7]^

The “Golden Triangle” program (ACEI/ARB + β-receptor blocker + aldosterone receptor antagonist) holds an important position in the treatment of CHF. Some new drugs, such as ivabradine hydrochloride, levosimendan, milrinone, etc., are the conventional treatment, but they cannot completely and effectively prevent the progression of heart failure. Recently, the traditional Chinese medicine (TCM) treatment for CHF has made great progress, and the 2014 China Heart Failure Prevention and Treatment Guidelines have recommended further research on TCM for heart failure.^[8]^

**Figure 1:**
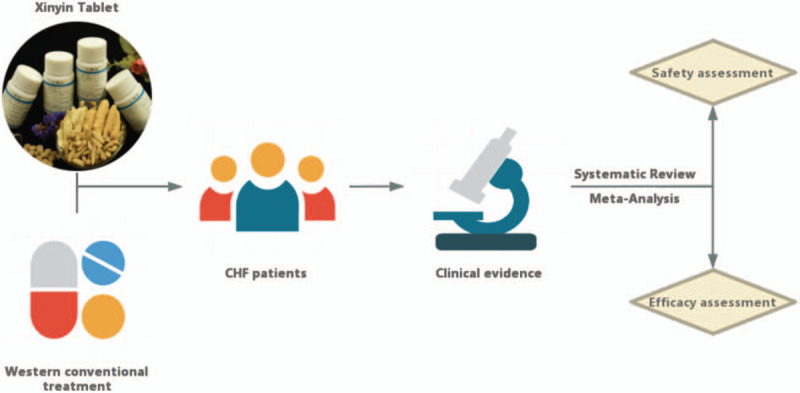
Work flow of this study.

Xinyin Tablet (XYT), composed of ginseng, astragalus, Ophiopogon japonicas, Schisandra, Motherwort, Maodongqing, Tinglizi, etc., was developed after numerous clinical and experimental studies at the First Affiliated Hospital of Guangzhou University of TCM. It was approved as a hospital preparation by the Guangdong Food and Drug Administration (approval number: “Yueyao Z20071178”), and has been in clinical use for several decades. Its remarkable efficacy has been widely recognized by clinicians and patients. However, the effectiveness and safety of XYT for CHF have not been comprehensively studied. Hence, this meta-analysis will be conducted to generalize the effectiveness and safety of XYT for CHF, which may benefit the design of future clinical trials and provide valuable references (Fig. 1).

## 2 Aim

This study is aimed to provide high-level evidence to prove the therapeutic effect of XYT on CHF, which may enhance the application of TCM.

## 3 Methods

This protocol complies with the Preferred Reporting Items for Systematic Review and Meta-Analysis Protocols guidelines.^[9]^ The protocol was registered on the International Platform of Registered Systematic Review and Meta-Analysis Protocols (INPLASY). The registration number was INPLASY202040105 (DOI number:

10.37766/inplasy2020.4.0105). This meta-analysis is based on secondary research of previously published data, so ethical approval and informed consent are not required.

### 3.1 Study design

This study will include clinical randomized controlled trials (RCTs) of XYT for CHF, regardless of the blinding method and language.

### 3.2 Eligibility criteria

The population-intervention-comparative-results-study design (PICOS) framework will be used to illustrate the eligibility criteria.

#### 3.2.1 Population

According to the diagnostic criteria,^[10–12]^ all patients (over 18 years of age) with the diagnosis of CHF regardless of their gender or race will be included in the study. Patients with other serious illnesses, such as acute cardiac insufficiency, obstructive cardiomyopathy, severe valvular disease, pulmonary embolism, etc., will be excluded.

#### 3.2.2 Intervention

The experimental group will include CHF patients treated with western conventional treatment combined with XYT.

#### 3.2.3 Comparator

The control group will include CHF patients treated with the same western conventional treatment as the intervention group.

#### 3.2.4 Outcome measures

##### 3.2.4.1 Main outcomes

1. The New York Heart Function classification;
2. The scores of quality of life and 6-min walk distance (6MWD).

##### 3.2.4.2 Additional outcome(s)

Secondary outcomes will include

1. TCM symptom scores;
2. The scores of quality of life;
3. Brain natriuretic peptide (BNP);
4. The cardiac color Doppler ultrasonographic indexes, such as left ventricular end-systolic volume (LVs), ejection fraction (EF) and fractional shortening (FS).

### 3.3 Information sources and search strategy

Four English electronic databases, including the Cochrane Library, PubMed, Web of Science (WOS) and Excerpt Medica Database (EmBase), and 4 Chinese electronic databases, including the Chinese Biomedical Literature Database (CBM), China National Knowledge Infrastructure (CNKI), China Scientific Journal Database (VIP) and Wanfang Database will be systematically searched for eligible studies, from their inception to September 2020. We will only select documents in English and Chinese. The search terms will be as follows: “chronic heart failure” or “chronic cardiac failure” or “heart failure” or “cardiac failure” or “heart decompensation” or “cardiac insufficiency” or “CHF” or “HF” and “Xinyin Tablet” or “XYT” or “Yangxinkang” or “Xinyin Pill”. Table 1 shows an example of the search strategy for PubMed database.

**Table 1:** Search strategy for PubMed.

Number	Terms
1	chronic heart failure
2	chronic cardiac failure
3	heart failure
4	cardiac failure
5	heart decompensation
6	cardiac insufficiency
7	CHF
8	HF
9	#1 OR #2 OR #3 OR #4 OR #5 OR #6 OR #7 OR #8
10	Xinyin Tablet
11	XYT
12	Yangxinkang
13	Xinyin Pill
14	#10 OR #11 OR #12 OR #13
15	#9 AND #14

### 3.4 Study selection and management

**Figure 2:**
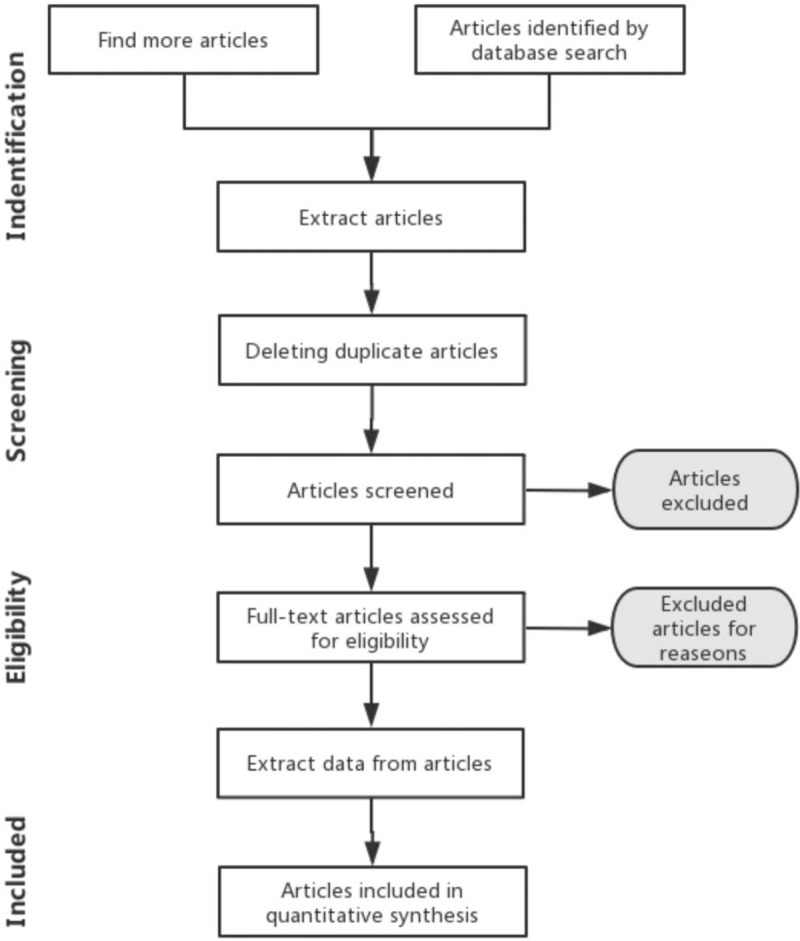
PRISMA compliant flow diagram.

The literature management and records search will be conducted using Endnote X9 software. Two authors (Xiaoming Dong and Jinhua Kang) will independently review and identify trials. Any disagreement will be resolved through discussion with a third reviewer (Mingyang Tian). The selection process of eligible studies is described in a flow diagram in Figure 2.

### 3.5 Data extraction and management

The data and relevant information, including diagnostic criteria, accompanying diseases, intervention and control group information, sample size, classification, observation time, outcomes, adverse effects, etc., will be collected using the Excel software. If the information provided in the study is unclear or erroneous, we will contact the corresponding author for further information.

### 3.6 Statistical analysis

Weighted mean difference (WMD) will be used to evaluate the extracted data of continuous variables, while the dichotomous variables will be analyzed by the odds ratio (OR). The confidence intervals (CI) will be set at 95%. The STATA 15.0 software will be used for heterogeneity testing. Chi-Squared test and inconsistency index statistics will be used to determine the statistical heterogeneity. The meta-analysis will be performed using the Mantel-Haenszel fixed model if *I*^2^ ≤ 50% and *P* ≥ .05. If *I*^2^ > 50% and *P* < .05, the heterogeneity will be analyzed through subgroup analysis, meta-regression, or descriptive analysis.^[13]^ The statistical heterogeneity, clinical heterogeneity, and methodological heterogeneity are 3 common forms of heterogeneity.^[14]^ Additionally, sensitivity analysis will be used to evaluate the potential sources of substantial heterogeneity. If more than 10 trials are included in the meta-analysis, the funnel plots will be used to estimate publication bias.^[15]^

### 3.7 Patient and public involvement

Patient and public involvement is not required in this study.

## 4 Discussion

In recent years, TCM has shown obvious advantages in the treatment of CHF, and its remarkable efficacy has been widely recognized by clinicians and patients.^[16]^ The 2014 China Heart Failure Prevention and Treatment Guidelines clearly recommended further research of TCM for CHF.^[8]^

The method of benefiting qi and nourishing yin is one of the main therapies in TCM for treating CHF,^[17]^ and both basic and clinical studies have shown remarkable therapeutic effects.^[18–20]^ XYT (formerly known as Yangxinkang) is a classic representative formula developed by the First Affiliated Hospital of Guangzhou University of TCM for the treatment of CHF. Modern pharmaceutical research has shown that Mao Dongqing^[21]^ has the effects of protecting heart and brain tissues, anti-coagulation, lowering blood pressure, anti-inflammatory, and enhancing immunity. Motherwort^[22]^ can inhibit myocardial hypertrophy, reduce the surface area of myocardial cells, and inhibit the activity of creatine kinase. The contractile response of vascular smooth muscle to vasoconstrictor substances is well established. Schisandra^[23]^ can improve myocardial ischemia-reperfusion injury, and has a strong antioxidant effect. Tinglizi^[24]^ can increase ventricular myocardial contractility, coronary flow and pumping blood function. XYT has been shown to reduce heart failure in animal models^[18,25–30]^ by lowering serum levels of tumor necrosis factor-α (TNF-α), interleukin-6 (IL-6), endothelin-1 (ET-1), angiotensin II (Ang II), aldosterone (ALD), improving cardiac ultrastructure, intervention of (Akt/AMPK)-mTOR signaling pathway, down-regulation of autophagy, reducing myocardial apoptosis, increasing left ventricular ejection fraction and shortening rate, delaying ventricular remodeling, inhibiting myocardial fibrosis, and improving cardiac function. Numerous clinical studies have reported that XYT can improve the systolic function of patients with asymptomatic heart failure,^[31]^ enhance NYHA heart function classification, quality of life score, exercise capacity, and reduce the incidence of ventricular arrhythmias.^[32–33]^ This study aims to provide a high-level evidence to establish the therapeutic effect of XYT on CHF, which may improve the symptoms and prognosis of patients, benefit the design of future clinical trials, and also provide valuable references. However, this study has some potential limitations. For example, in the western conventional treatment, the manufacturers, combination and dosage of different drugs, and the degree of heart failure are not uniform, which may result in significant heterogeneity. Besides, the discrepancies in the clinical application of TCM worldwide and the limitation of search language may lead to a geographical bias.

## Acknowledgments

We are grateful to Professor Licheng Zhao from Guangzhou University of Chinese Medicine for his inspiring guidance to this work. We also thank Miss Jingyi Xu from South China Normal University for her critical revision of the article.

## Author contributions

**Conceptualization:** Qingqing Liu, Xi Huang, Huili Liao, Wenjie Long, Zhongqi Yang.

**Data curation:** Xiaoming dong Dong, Jinhua Kang.

**Funding acquisition:** Jianhong Liu.

**Investigation:** Jianhong Liu.

**Methodology:** Mingyang Tian, Yan Ling.

**Project administration:** Mingyang Tian, Xiaoming dong Dong, Jinhua Kang, Jianhong Liu.

**Resources:** Mingyang Tian, Xiaoming dong Dong, Jinhua Kang.

**Software:** Xiaoming dong Dong, Jinhua Kang.

**Supervision:** Huili Liao.

**Validation:** Yan Ling.

**Visualization:** Yan Ling.

**Writing – original draft:** Qingqing Liu, Xi Huang.

**Writing – review & editing:** Wenjie Long, Zhongqi Yang.
